# Exploring research engagement among nurses in a Magnet®-recognized cancer center: An analysis of knowledge, attitudes, practices, and influencing factors

**DOI:** 10.1016/j.apjon.2024.100545

**Published:** 2024-06-20

**Authors:** Amanda Drury, Kristen L. Fessele, Piera Robson, Ethel Law, Margaret Barton-Burke, Bridgette Thom

**Affiliations:** a School of Nursing, Psychotherapy and Community Health, Dublin City University, Dublin, Ireland; b Memorial Sloan Kettering Office of Nursing Research, New York, NY, USA; c Department of Nursing, Memorial Sloan Kettering Cancer Center, New York, NY, USA; d Gastric and Mixed Tumor Service, Department of Surgery, Memorial Sloan Kettering Cancer Center, New York, NY, USA; e School of Social Work, University of North Carolina, Chapel Hill, NC, USA

**Keywords:** Neoplasms, Nurses, Research, Knowledge, Attitude, Practice

## Abstract

**Objective:**

Despite the significance of research in nursing practice and its role in enhancing the quality of life for cancer patients, nurses report limited opportunities to engage with research. Known barriers include limited organizational support, a lack of time, resources, and knowledgeable colleagues/mentors. The study aims to determine research knowledge, attitudes, and practices among cancer nurses and understand factors influencing nurses’ involvement in research.

**Methods:**

Registered nurses responded to a cross-sectional questionnaire. Data were collected using a modified version of the Nursing Research Knowledge, Attitudes, and Practices survey and the Barriers to Nurses’ Participation in Research Questionnaire.

**Results:**

Three hundred and sixty-six nurses responded, of whom 15% had previously been involved in research. Nurses reported moderate to high research knowledge (μ= 1.72), attitudes (μ= 1.92), and practice (μ= 1.79) scores. The most common barriers to engagement with research included a lack of time (78.0%), lack of incentive or reward (61.4%), lack of knowledge or skills (54.2%), lack of training opportunities (51.4%), and lack of experienced nursing research mentors (44.8%). Holding a specialist, advanced practice, or administrative role, and a postgraduate qualification were associated with higher scores on the knowledge, attitudes and practice subscales. Previous experience of conducting research was associated with higher knowledge and attitudes scores. Previous experience of authoring a paper and submitting a grant were associated with higher knowledge scores.

**Conclusions:**

The study reveals a moderate–to-high level of knowledge, attitudes, and practice regarding research among nurses, but low engagement. Barriers include lack of time, incentive, knowledge, training, and mentorship. Nevertheless, attitude scores, reflecting nurses’ willingness to engage in research were high on average. The findings highlight the potentially modifiable nature of barriers to research engagement. Increasing capacity for nursing research may be achieved through investment in research support and training to overcome barriers to research, which may discourage nurses engagement.

## **Introduction**

Research is a core competence in nursing and integral to licensing, certification, and accreditation requirements; as a result, there is an expectation that nurses should be engaging with research in some capacity in their practice.^1^, ^2^, ^3^, ^4^, ^5^, ^6^, ^7^, ^8^ The Magnet® Recognition Program, for example, requires institutions seeking designation to base practice on evidence, ensure nurses apply research findings to practice, and support nursing research endeavors.^7^^,^^9^ Despite this, nurses report limited opportunities to engage with research, either through clinical application or the actual conduct and dissemination of nursing-led research.^10^, ^11^, ^12^

The role of cancer nursing research in advancing understanding of the illness experiences of people living with and after cancer has been significant, as has its role in developing and implementing interventions that enhance quality of life for those living with and after cancer.^13^, ^14^, ^15^, ^16^ However, promoting interest and engagement in research among cancer nurses remains a significant challenge to future nursing research, as research education within undergraduate and postgraduate nursing programs is often segregated from clinical theory.^3^^,^^17^ Laterally, perceptions of nursing roles in research as supporting rather than leading research remain pervasive.^18^^,^^19^ This confluence of systemic barriers to building capacity for cancer nursing research may serve to undermine advancements in the development and implementation of specialist, advanced practice and nurse scientist roles, which require ongoing leadership and engagement in research.^20^, ^21^, ^22^

In general, barriers to the utilization and conduct of research among nurses include limited organizational support and a lack of time, resources, and/or knowledgeable colleagues/mentors.^20^^,^^21^^,^^23^, ^24^, ^25^, ^26^ These barriers have been shown to negatively impact nurses' satisfaction with engaging with research.^10^ Nurses' participation in research relates to both their ability to access the resources, tools, and time needed to conduct research.^23^ Furthermore, the perceived relevance of research to one's clinical role has also been identified as a latent barrier to nurses' involvement in research.^23^ These findings underscore previous research that suggests nurses' knowledge regarding research and willingness to participate in research activities relate to their organizational exposure to trained research mentors and internal experts, support from nursing administration (through allocated time for training, protected time to conduct research, and development of a research infrastructure), and demonstrated application of research findings in clinical practice.^5^^,^^23^, ^24^, ^25^

A recent synthesis of literature relating to the engagement of nurses in research highlighted five key factors in implementing a successful nursing research program: access to a research infrastructure, support from leadership, developing institutional research priorities and interests, diversifying educational strategies, and leveraging available resources and established networks.^11^ The authors suggest a needs assessment to be a critical first step in the multifaceted approach required to support nurses in engaging with, conducting, and disseminating research. The current study was undertaken as a formative step to inform the development of programs to enhance the engagement of nurses with research within their roles. Garnering an understanding of specific strengths, weaknesses, facilitators, and barriers to research within clinical practice roles is an essential first step to ensure that future interventions are robust and fit for purpose.^9^^,^^27^^,^^28^ Therefore, the purpose of this study was to determine research knowledge, attitudes, and practices among nurses employed in a large Magnet®-recognized cancer center and understand factors influencing nurses’ involvement in research.

## **Methods**

### Design

A cross-sectional survey study was undertaken with registered nurses with a current nursing license working at Memorial Sloan Kettering Cancer Center.

### Data collection

A modified version of the Nursing Research Knowledge, Attitudes, and Practices survey^29^ was used to collect data on three subscales related to (1) research *knowledge*, (2) willingness to engage in or perform research (*attitudes*), and (3) perceived ability to perform research (*practice*) related to 23 research activities. The scale was modified with the permission of the instrument designer due to concerns from institutional leadership regarding the survey burden on potential participants. To minimize the burden on participants, the instrument was revised to condense instrument length, focusing on items related to the types of nursing inquiry that are conducted in the institution and those which aligned with strategic research development within the organization. The Nursing Research Knowledge, Attitudes, and Practices items are rated on a three-point ranking scale, with higher scores reflecting more positive knowledge, attitudes and practice. The Nursing Research Knowledge, Attitudes, and Practices Questionnaire was developed based on the Iowa Model for Evidence-Based Practice,^30^, ^31^, ^32^ and has demonstrated acceptable test-retest reliability (knowledge *r* = 0.83; attitude *r* = 0.81; practice *r* = 0.77) and internal consistency (knowledge α = 0.94; attitude α = 0.97; practice α = 0.93).^29^

The Barriers to Nurses’ Participation in Research Questionnaire^23^ was used to assess barriers to nursing research via two subscales: research resources and personal relevance of research, and a standalone question relating to time. The Barriers to Nurses’ Participation in Research Questionnaire items are rated on a five-point ranking scale, with higher scores reflecting higher levels of perceived barriers to participation in research. The Barriers to Nurses’ Participation in Research Questionnaire has been psychometrically tested and demonstrated acceptable internal consistency (resources α = 0.79; relevance α = 0.74).^23^

The questionnaire was administered anonymously via Research Electronic Data Capture (REDcap).^33^ An email introducing the study was distributed to potential participants via the organization's all-nursing email distribution list. An eligibility screening question was used to ensure only nurses employed by the organization complete the survey.

### Data analysis

Variables assessing nurses’ knowledge, attitudes, practice and perceived barriers to research engagement were descriptively analyzed using frequencies. Instrument subscales were scored according to author guidelines, and measures of central tendency were generated for each of the Nursing Research Knowledge, Attitudes, and Practices and Barriers to Nurses’ Participation in Research Questionnaire subscales. Demographic factors influencing knowledge, attitudes, practice and barriers to research were assessed using inferential statistical tests, including chi-square and independent sample *t*-tests. Relationships between the subscales of the Nursing Research Knowledge, Attitudes, and Practices and Barriers to Nurses’ Participation in Research were tested via Spearman correlations.

### Ethical considerations

Potential respondents were assured that participation in the study was voluntary and anonymous, and that return of a completed questionnaire implied consent. The study received approval from the Institutional Review Board (IRB No. X18-003).

## **Results**

### Demographic characteristics

A total of 366 nurses took part in this study. Participants had an average of 15.1 years of experience (SD = 11.3, range = 0–46) and worked in in-patient (23.0%), out-patient (53.3%), perioperative (16.9%), pediatric (1.9%), and other (4.9%) settings. Participants were primarily clinical nurses (71.0%), with the majority working at levels I or II on the institution's clinical ladder, indicating less than 18 months or up to 3 years of nursing experience, respectively (42.5%). The remaining participants were Nurse Practitioners (11.5%), Clinical Research Nurses (6.0%), Clinical Nurse Specialists/Nursing Professional Development Specialist (3.8%), administrative (3.6%), or other (2.7%) nursing staff. At the time of the study, 19.6% of the participants were enrolled in further education. The majority of participants had completed a bachelor’s degree at the highest level of education (64.7%), and more than one-quarter had completed a master’s degree (27.4%). Over two-thirds (68.8%) of the sample expressed a desire to achieve higher levels of education, of whom the majority reported desiring master’s-level education (55.4%), with 29.9% and 12.4% of participants desired DNP or EdD/PhD-level education, respectively. About one-third (29.9%) of respondents had achieved their desired level of education (Table 1), with the majority of these holding a bachelor’s degree (50.5%) or master’s degree (36.7%).

**Table 1:** Sample characteristics.

Variable	*n*	%
Years practicing as a registered nurse	Mean: 15.1	SD: 11.3
**Area of practice (*n* = 366)**		
In-patient	84	23.0
Outpatient	195	53.3
Perioperative	62	16.9
Pediatrics	7	1.9
Other	18	4.9
**Current role (*n* = 365)**		
Clinical Nurse Grade I–II	155	42.5
Clinical Nurse Grade III–IV	104	28.5
Clinical Research Nurse	22	6.0
CNS/NPDS	14	3.8
CRNA	5	1.4
Nurse practitioner	42	11.5
Administration (nurse leader, NPCNS coordinator, Director)	13	3.6
Other	10	2.7
**Highest nursing degree (*n* = 365)**		
Diploma/Associates	19	5.2
Bachelors	236	64.7
Masters	100	27.4
DNP	7	1.9
PhD/EdD	3	0.8
**Currently studying for MSc, DNP, PhD/EdD (*n* = 366)**	72	19.7
**Desires higher degree (*n* = 365)**		
Did not express desire	5	1.4
Expresses desire for higher level of education	251	68.8
Has achieved highest desired level of education	109	29.9
**Desired highest nursing degree (*n* = 251)**		
Bachelors	6	2.4
Masters	139	55.4
DNP	75	29.9
PhD/EdD	31	12.4

CNS, Clinical Nurse Specialist; NPDS, Nursing Professional Development Specialists; CRNA, Certified Registered Nurse Anesthetist; DNP, Doctor of Nursing Practice; MSc, Master of Science; EdD, Doctor of Education; SD, standard deviation.

### Previous involvement in research

There was limited reported involvement in research (14.8% of the sample): of these, 55.6% engaged as a principal investigator or co-principal investigator, and 68.5% as an investigator (Table 2). Those who had been involved in research previously reported having been involved in retrospective (40.7%), prospective (20.4%), quality improvement (37.0%), non-therapeutic (25.9%), or therapeutic studies (44.4%) (response options were not mutually exclusive).

**Table 2:** Previous involvement in research.

Variable	*n*	%
**Has been involved in research (*n* = 366)**	54	14.8
**Has been involved in research as (*n* = 54)**		
a PI/Co-PI	30	55.6
as an investigator	37	68.5
**Types of studies involved in (*n* = 54)**		
Retrospective study	22	40.7
Prospective study	11	20.4
Quality improvement study	20	37.0
Non-therapeutic study	14	25.9
Therapeutic study	24	44.4
Other study	3	5.6
**Previous experience of research activities (*n* = 366)**		
Submitted a research abstract to a professional conference	82	22.4
Abstract accepted for a poster presentation	69	84.1
Abstract accepted for a podium or panel presentation	39	47.6
Authored or co-authored a peer-reviewed publication	71	19.4
Submitted a grant to conduct a research study	22	6.0
**Types of publications authored (*n* = 366)**		
Primary research study	29	7.9
Secondary analysis	4	1.1
Quality improvement project	13	3.5
Clinically focused article	37	10.1

PI, principal investigator.

Six percent of participants (*n* = 22) had been involved in the submission of a grant. Eighty-two participants had previously submitted a research abstract (22.4%), of whom 84.1% reported the abstract had been accepted for poster presentation and 47.6% accepted as a podium presentation. A similar proportion of participants had been involved in the authorship of peer-reviewed publications (19.4%, *n* = 71). Of those who had reported involvement in the authorship of peer-reviewed publications, 40.8% reported authorship of manuscripts focused on primary research, and 52.1% reported authorship of manuscripts with a clinical focus.

### Nursing research, knowledge, attitudes and practice

Participants were invited to rate their knowledge, attitudes and practice of 23 research activities. The mean knowledge score for this sample was 1.72 (SD = 0.49, range = 0.09–3.00). The most common items that nurses expressed high knowledge of included the identification of clinical problems (44.4%, *n* = 131), identifying new information in the literature (32.6%, *n* = 95), gathering relevant literature (29.1%, *n* = 85), getting support from your nurse leader (23.2%, *n* = 66), and preparing a poster presentation (21.8%, *n* = 62). The items for which participants were most likely to report low levels of knowledge were submitting a proposal for grant funding (71.3%, *n =* 204), submitting a proposal for IRB review (63.4%, *n =* 184), setting up procedures for the study (55.8%, *n =* 159), finding the right data collection instrument (51.4%, *n =* 148), and collaborating with the Office of Nursing Research (50.7%, *n* = 145) (Table 3).

**Table 3:** Participants’ self-rated knowledge, attitudes and practice related to research.

	Knowledge						Attitudes						Practice					
	Low		Moderate		High		Low		Moderate		High		Low		Moderate		High	
	*n*	%	*n*	%	*n*	%	*n*	%	*n*	%	*n*	%	*n*	%	*n*	%	*n*	%
Identifying a clinical problem from practice experience	15	5.1	149	50.5	131	44.4	11	3.7	136	46.3	147	50.0	26	9.0	137	47.2	127	43.8
Identifying new information in the literature with implications for current practice	35	12.0	161	55.3	95	32.6	20	6.9	141	49.0	127	44.1	28	9.9	151	53.4	104	36.7
Gathering relevant research literature	49	16.8	158	54.1	85	29.1	36	12.3	155	53.1	101	34.6	37	12.8	161	55.5	92	31.7
Critiquing research literature for use in practice	88	30.0	146	49.8	59	20.1	72	24.8	139	47.9	79	27.2	74	26.1	142	50.0	68	23.9
Determining if there is a sufficient research base for practice change	79	27.1	168	57.5	45	15.4	51	17.5	158	54.3	82	28.2	61	21.6	158	55.8	64	22.6
Developing/identifying a theoretical framework for study	121	41.7	146	50.3	23	7.9	79	27.4	151	52.4	58	20.1	97	33.7	150	52.1	41	14.2
Formulating research questions	81	27.9	167	57.6	42	14.5	54	18.6	160	55.2	76	26.2	70	24.6	167	58.6	48	16.8
Developing a proposal to study a research question	131	45.2	130	44.8	29	10.0	83	28.6	144	49.7	63	21.7	103	35.8	145	50.3	40	13.9
Finding the right data collection instrument	148	51.4	121	42.0	19	6.6	85	29.7	151	52.8	50	17.5	117	41.5	133	47.2	32	11.3
Submitting a proposal to the institutional review board	184	63.4	87	30.0	19	6.6	106	37.1	130	45.5	50	17.5	139	48.8	113	39.6	33	11.6
Submitting a proposal for grant funding	204	71.3	71	24.8	11	3.8	119	41.9	121	42.6	44	15.5	148	53.2	106	38.1	24	8.6
Getting support from your nurse leader to do the study	86	30.3	132	46.5	66	23.2	67	23.7	142	50.2	74	26.1	82	29.4	132	47.3	65	23.3
Getting peer support to do the study	85	29.5	159	55.2	44	15.3	62	21.6	165	57.5	60	20.9	73	26.1	156	55.7	51	18.2
Building your study team	121	42.9	131	46.5	30	10.6	84	29.9	149	53.0	48	17.1	100	36.0	140	50.4	38	13.7
Collaborating with the Office of Nursing Research	145	50.7	112	39.2	29	10.1	85	30.0	139	49.1	59	20.8	104	37.0	138	49.1	39	13.9
Setting up procedures (methods) for the study	159	55.8	110	38.6	16	5.6	97	34.0	144	50.5	44	15.4	120	42.3	135	47.5	29	10.2
Collecting data	102	35.4	152	52.8	34	11.8	63	22.0	164	57.1	60	20.9	85	29.9	152	53.5	47	16.5
Entering the data into a database	130	45.6	115	40.4	40	14.0	82	28.8	139	48.8	64	22.5	103	36.1	128	44.9	54	18.9
Analyzing the data	124	43.4	139	48.6	23	8.0	96	33.4	134	46.7	57	19.9	115	40.1	130	45.3	42	14.6
Drawing conclusions from the study results	106	37.3	148	52.1	30	10.6	79	27.9	143	50.5	61	21.6	92	33.1	145	52.2	41	14.7
Writing up the results for publication	138	48.6	125	44.0	21	7.4	97	34.2	134	47.2	53	18.7	117	41.3	131	46.3	35	12.4
Presenting the results orally (podium presentation)	109	38.4	120	42.3	55	19.4	87	30.7	120	42.4	76	26.9	92	32.6	122	43.3	68	24.1
Preparing a poster presentation	87	30.5	136	47.7	62	21.8	63	22.3	135	47.7	85	30.0	67	23.7	140	49.5	76	26.9

With regard to attitudes, the average attitude score was 1.92 (SD = 0.59, range = 0.13–3.00). Participants were most likely to express a willingness to engage with activities including identification of a clinical problem (50.0%, *n* = 147), identifying new information in the literature (44.1%, *n* = 127), gathering relevant literature (34.6%, *n* = 101), preparing a poster presentation (30%, *n* = 85), and determining if there is a sufficient research base for practice change (28.2%, *n* = 82). The activities that participants were least willing to engage with were submitting a proposal for grant funding (41.9%, *n* = 119), submitting a proposal for IRB review (37.1%, *n* = 106), writing results for publication (34.2%, *n* = 97), setting up procedures for the study (34.0%, *n* = 97), and analyzing data (33.4%, *n* = 96) (Table 3).

Finally, mean practice scores were 1.79 (SD = 0.56, range = 0.09–3.00). Participants were most likely to perceive high levels of ability with the identification of a clinical problem (43.8%, *n* = 127), identifying new information in the literature (36.7%, *n* = 104), gathering relevant literature (31.7%, *n* = 92), preparing a poster presentation (26.9%, *n* = 76), and presenting the results of a study orally (24.1%, *n* = 68). The items for which participants identified the lowest level of ability to perform included submitting a proposal for grant funding (53.2%, *n* = 148), submitting a proposal for IRB review (48.8%, *n* = 139), setting up study methods (42.3%, *n* = 120), and finding the right data collection instrument (41.5%, *n* = 117), and writing the results up for publication (41.3%, *n* = 117) (Table 3).

### Barriers to nurses’ participation in research

90.9% of participants (*n* = 333) identified one or more barriers to research in their practice, with a mean of 4.8 barriers reported by the sample (SD = 3.3, range = 0–14). The mean score for the research resources scale was 3.13 (SD = 0.82, range = 1–5) for personal relevance of research was 2.33 (SD = 0.64, range = 1–4.83) and time was 4.03 (SD = 1.01, range = 1–5). The most common barriers identified were a lack of time (78.0%, *n* = 262), a lack of incentive or reward for nurses to be involved in research (61.4%, *n* = 205), lack of knowledge or skills (54.2% *n* = 182), lack of training opportunities (51.4%, *n* = 171), and lack of experienced nursing research mentors (44.8%, *n* = 150) (Table 4).

**Table 4:** Barriers to nurses’ participation in research.

	Strongly disagree		Disagree		Neither agree nor disagree		Agree		Strongly agree	
	*n*	%	*n*	%	*n*	%	*n*	%	*n*	%
Lack of leadership support (*n* = 335)	41	12.2	113	33.7	102	30.4	64	19.1	15	4.5
Lack of institutional nursing research infrastructure (*n* = 332)	31	9.3	114	34.3	107	32.2	63	19.0	17	5.1
Lack experienced nursing research mentors (*n* = 335)	23	6.9	70	20.9	92	27.5	125	37.3	25	7.5
Lack of accessibility to the nursing research council (*n* = 336)	20	6.0	66	19.6	111	33.0	113	33.6	26	7.7
Lack of resources to facilitate nursing research (*n* = 335)	23	6.9	93	27.8	93	27.8	97	29.0	29	8.7
Lack of research training opportunities (*n* = 333)	19	5.7	64	19.2	79	23.7	133	39.9	38	11.4
Lack of incentive/reward for nurses to do research (*n* = 334)	18	5.4	44	13.2	67	20.1	142	42.5	63	18.9
Lack of research knowledge or skills (*n* = 336)	21	6.3	64	19.0	69	20.5	140	41.7	42	12.5
Lack of time to research (*n* = 336)	10	3.0	22	6.5	42	12.5	134	39.9	128	38.1
I do not have ideas for research project topics (*n* = 337)	30	8.9	109	32.3	86	25.5	93	27.6	19	5.6
Research is not very interesting or valuable to me (*n* = 333)	80	24.0	162	48.6	55	16.5	25	7.5	11	3.3
Research is not relevant to nursing practice (*n* = 336)	203	60.4	117	34.8	9	2.7	6	1.8	1	0.3
My training/education do not qualify me to conduct research (*n* = 336)	74	22.0	154	45.8	78	23.2	27	8.0	3	0.9
I feel intimidated by research (*n* = 336)	48	14.3	79	23.5	68	20.2	114	33.9	27	8.0
Nursing research is not my job (*n* = 336)	72	21.4	134	39.9	81	24.1	37	11.0	12	3.6

### Factors influencing nurses’ knowledge, attitudes, practice and barriers to research

The results of *t*-tests to identify factors associated with Nursing Research Knowledge, Attitudes, and Practices Questionnaire subscales and Barriers to Nurses’ Participation in Research Questionnaire subscales are presented in Table 5, Table 6, respectively. The results of Spearman correlations to establish relationships between the subscales of the Nursing Research Knowledge, Attitudes, and Practices and Barriers to Nurses’ Participation in Research are presented in Table 7.

**Table 5:** Factors associated with participants’ self-rated knowledge, attitudes and practice related to research.

	Knowledge					Attitudes					Practice				
	*n*	Mean	SD	*t*	*P*	*n*	Mean	SD	*t*	*P*	*n*	Mean	SD	*t*	*P*
**Role**															
Clinical nurse	207	1.66	0.50	−2.773	0.006	207	1.86	0.59	−2.770	0.006	207	1.74	0.56	−2.614	0.009
CRN/CNS/CRNA/NP/Admin/Other	89	1.84	0.47			89	2.06	0.56			89	1.92	0.54		
**Degree**															
Diploma/Associate/Bachelors	201	1.62	0.48	−5.042	≤ 0.005	201	1.84	0.57	−3.349	0.001	201	1.71	0.54	−3.937	≤ 0.005
Masters/PhD/DNP	95	1.92	0.47			95	2.08	0.59			95	1.97	0.55		
**Research conduct**															
None	250	1.68	0.47	−3.028	0.003	250	1.89	0.57	−2.173	0.031	250	1.77	0.53	−1.923	0.055
Involvement in research as a PI/Co-PI/Investigator	47	1.91	0.59			47	2.09	0.65			47	1.94	0.65		
**Authorship**															
No	230	1.68	0.47	−2.705	0.007	230	1.91	0.59	−1.035	0.301	230	1.78	0.55	−0.886	0.376
Yes	64	1.86	0.55			64	1.99	0.58			64	1.85	0.59		
**Grant**															
No	273	1.69	0.48	−3.372	0.001	273	1.91	0.58	−1.791	0.074	273	1.78	0.55	−1.962	0.051
Yes	21	2.06	0.53			21	2.14	0.59			21	2.02	0.65		

CRN, Certification and Recertification for Nurse; CNS, Clinical Nurse Specialist; CRNA, Certified Registered Nurse Anesthetist; NP, Nurse Practitioner; DNP, Doctor of Nursing Practice; PI, principal investigator; SD, standard deviation.

**Table 6:** Factors associated with barriers to research for nurses.

	Research resources					Personal relevance of research					Time				
	*n*	Mean	SD	*t*	*P*	*n*	Mean	SD	*t*	*P*	*n*	Mean	SD	*t*	*P*
**Role**															
Clinical nurse	234	3.10	0.86	−1.119	0.264	234	2.40	0.63	3.109	0.002	233	3.97	1.09	−2.03	0.043
CRN/CNS/CRNA/NP/Admin/ Other	102	3.20	0.72			102	2.17	0.63			102	4.19	0.83		
**Degree**															
Diploma/Associate/Bachelors	230	3.05	0.87	−2.704	0.007	230	2.41	0.64	3.286	0.001	229	3.93	1.08	−3.015	0.003
Masters/PhD/DNP	106	3.30	0.69			106	2.17	0.61			106	4.25	0.83		
**Research conduct**															
None	285	3.08	0.82	−2.644	0.009	285	2.38	0.63	3.331	0.001	284	3.97	1.05	−2.865	0.004
Involvement in research as a PI/Co-PI/Investigator	52	3.40	0.78			52	2.07	0.64			52	4.40	0.75		
**Authorship**															
No	265	3.08	0.83	−2.153	0.032	265	2.40	0.63	3.797	≤ 0.005	264	3.92	1.05	−3.774	≤ 0.005
Yes	69	3.31	0.77			69	2.08	0.62			69	4.43	0.76		
**Grant**															
No	313	3.10	0.83	−2.044	0.042	313	2.35	0.63	2.407	0.017	312	4.00	1.03	−2.069	0.039
Yes	21	3.48	0.62			21	2.01	0.72			21	4.48	0.75		

CRN, Certification and Recertification for Nurse; CNS, Clinical Nurse Specialist; CRNA, Certified Registered Nurse Anesthetist; NP, Nurse Practitioner; DNP, Doctor of Nursing Practice; PI, principal investigator; SD, standard deviation.

**Table 7:** Spearman's correlations between knowledge, attitudes, practice and barriers to research for nurses.

	Years of RN practice		Knowledge		Attitudes		Practice		Research resources		Personal relevance		Time	
	*r*	*P*	*r*	*P*	*r*	*P*	*r*	*P*	*r*	*P*	*r*	*P*	*r*	*P*
Years of RN practice			−0.125∗	0.031	−0.160∗∗	0.006	−0.173∗∗	0.003	0.036	0.507	0.006	0.907	0.110∗	0.044
Knowledge	−0.125∗	0.031			0.707∗∗	≤ 0.005	0.844∗∗	≤ 0.005	−0.111	0.056	−0.441∗∗	≤0.005	−0.004	0.951
Attitudes	−0.160∗∗	0.006	0.707∗∗	≤ 0.005			0.832∗∗	≤ 0.005	0.028	0.633	−0.440∗∗	≤ 0.005	0.046	0.434
Practice	−0.173∗∗	0.003	0.844∗∗	≤ 0.005	0.832∗∗	≤ 0.005			−0.115∗	0.048	−0.418∗∗	≤ 0.005	−0.035	0.543
Research resources	0.036	0.507	−0.111	0.056	0.028	0.633	−0.115∗	0.048			0.044	0.416	0.451∗∗	≤ 0.005
Personal relevance	0.006	0.907	−0.441∗∗	≤ 0.005	−0.440∗∗	≤ 0.005	−0.418∗∗	≤ 0.005	0.044	0.416			−0.060	0.277
Time	0.110∗	0.044	−0.004	0.951	0.046	0.434	−0.035	0.543	0.451∗∗	≤ 0.005	−0.060	0.277		

∗*P* ≤ 0.05, ∗∗*P* ≤ 0.005. RN, Registered Nurse.

Higher research knowledge scores were associated with holding a specialist, advanced practice, or administrative role (*P* = 0.006), a postgraduate qualification (*P* < 0.001), previous experience of conducting research (*P* = 0.003), authoring a paper (*P* = 0.007), and submitting a grant (*P* = 0.001). Higher scores on the attitudes and practice subscales were associated with holding a specialist, advanced practice or administrative role, and a postgraduate qualification only (*P* < 0.05). Previous experience with research, authorship, or grantsmanship were not associated with differences in scores for research attitudes and practice (*P* > 0.05) (Table 5).

Those who had higher scores on the research barriers resource subscale were more likely to have postgraduate education (*P* = 0.007), and have conducted research previously (*P* = 0.009), been involved in the authorship of a manuscript (*P* = 0.032), and have submitted a grant (*P* = 0.042). Those with postgraduate education, working in specialist, leadership, and administrative roles, and who had been involved in the conduct of research, authorship of a paper, or submission of a grant were more likely to indicate research was relevant to them and more likely to report having less time available to conduct research (*P* < 0.05) (Table 6).

Years practicing as a registered nurse was positively correlated with participants’ perceptions of time available to conduct research (*P* = 0.044), such that those with greater experience were more likely to report having time available to conduct research. Years practicing as a registered nurse were negatively correlated with knowledge (*P* = 0.031), attitudes (*P* = 0.006) and practice (*P* = 0.003) subscales, such that nurses with greater experience were less likely to report higher levels of knowledge, attitudes and practice toward research on these subscales; however, the correlation for each subscale was weak (range: −0.124–0.173) (Table 7).

Research resource subscale scores were weakly correlated with research practice (*r* = −0.115, *P* = 0.048), such that those who reported greater barriers to research as a result of resources were less likely to express a willingness to perform research activities. Participants who reported that research was less relevant to them on the personal relevance subscale of the questionnaire were more likely to express lower knowledge (*P* < 0.001), attitudes (*P* < 0.001), and willingness to perform research (*P* < 0.001). Time was not correlated with research knowledge, attitudes, or practice scores (*P* > 0.05). However, having less time to conduct research was correlated with a greater perception of resource-related barriers to research (*P* < 0.001) (Table 7).

Nurses knowledge, attitudes and practice were all correlated (*P* < 0.005), demonstrating that those with greater self-perceived knowledge were more likely to have more positive attitudes toward research and a greater willingness to perform research activities. Likewise, those with more positive attitudes reported a greater willingness to perform research activities (Table 7).

## Discussion

While this study has highlighted moderate to high levels of knowledge, attitudes, and practice regarding research among nurses; yet, within this sample, there were relatively low levels of engagement, with less than 15% reporting involvement in research. Lack of time, incentive, knowledge, training and mentorship were identified as the most significant barriers to engagement in research. Nevertheless, a large proportion of participants expressed a desire to engage in further education involving research, at both the master’s and doctoral levels. Furthermore, attitude scores, reflecting nurses’ willingness to engage in research, were higher on average compared to knowledge and practice scores. Collectively, when considered alongside the potentially modifiable nature of barriers to research engagement, the findings suggest that there is an appetite among nurses to engage with research, where appropriate resourcing and training are provided. However, to overcome these barriers, investment is needed to address education, research culture, and resourcing to support nursing research.

The results of this study align with previous research regarding factors influencing knowledge, attitudes and practice related to research.^34^^,^^35^ Specifically, having higher levels of education and holding a specialist, advanced practice or administrative role were associated with higher levels of research knowledge and greater willingness and perceived ability to perform research. The majority of participants in this study reported that bachelor’s-level was their highest educational achievement at the time of the study, reflecting that two-fifths of the sample had between 18 months and three years of clinical experience. Previous research suggests that the completion of master's-level education has a positive impact on graduate nurses'; including greater utilization of research to inform clinical practice, and an increased likelihood of engaging in research-related tasks.^36^ While the majority of participants reported lower levels of academic education in nursing, an almost equivalent proportion expressed a desire to progress to masters- and doctoral-level education. This positive trend presents an opportunity to build capacity for nursing research, particularly when considered alongside findings that participants express a desire and positive attitude towards conducting research. However, the means by which further education is supported and by which research is integrated into the nursing curriculum within the taught programs is critical.

Despite positive findings regarding perceived knowledge, attitudes and practice regarding research, the proportion of participants who had experience of research leadership or involvement in supporting research leadership activities was relatively low within this study. Just over one-tenth of participants had experience as a principal investigator or co-investigator. Less than one-fifth of participants reported prior involvement in the preparation and submission of a research grant (6.0%), or the preparation and submission of a manuscript for peer-review (19.4%). These findings are reflected in participants' responses to the knowledge, attitudes and practice questionnaire, with a large proportion of participants reporting low or moderate willingness to engage with the preparation of a research grant and the preparation of a manuscript for publication. Predominantly, those who had experience of research dissemination reported that this was limited to poster presentation. These results lend significance to the assertion that nurses’ roles in clinical research may more often be perceived as supporting research.^3^^,^^18^^,^^19^ Leveraging interest and willingness among nurses to engage in research will be crucial for capacity-building in the future. Role modeling and peer mentorship, as well as expert mentorship, may support the scaling of capacity for nursing research, particularly among early-career nurses, and nurses educated to bachelor-level. However, in order to foster positive research cultures in clinical practice, organizations must provide clear pathways to securing research mentorship within the clinical environment and through academic partnerships.

While this study presents tentatively positive results regarding nurses' willingness to engage in research, and knowledge and perceived ability to engage in research, there remain consistent barriers to engagement in research, including resourcing, incentivization, and supports to foster a more positive research culture. Subgroups who were less likely to engage in research, including those with lower levels of education and working within clinical roles, were more likely to report lower levels of knowledge, attitudes and practice regarding research, but less likely to report barriers to conducting research. Furthermore, nurses working in specialist, advanced practice, or administrative roles were more likely to report more positive knowledge, attitudes and practice toward research, nurses with more years of experience reported less positive knowledge, attitudes and practice toward research. These findings may be reflective of the distribution of the sample; more than 70% of participants worked in clinical nurse roles. Working as a clinical nurse was associated with a lower likelihood of recognizing research as part of one's role.

### Implications for nursing practice and research

Without strategies to ameliorate barriers to research practice, there is little that can be achieved through education and mentorship to support enhanced capacity for clinical nursing research. Investment in Nursing Research Fellowships offer significant opportunities to build capacity, targeting aspects of research practice which nurses in this study reported lower levels of knowledge and confidence with, including preparation of the research protocol, submission for IRB review, and analysis and dissemination of results via peer-reviewed manuscripts.^37^ While the formal outcomes of the Mazzella Ebstein, Barton-Burke^37^ Nursing Research Fellowship focused on program completion and internal dissemination, the results of this study demonstrate significant impact, extending beyond the vision for the program, with 21 manuscripts prepared from 15 IRB studies conducted within the Nursing Research Fellowship cohort to date. Other strategies which may address resource-based challenges include seed funding, buyout of clinical time, and development of formal clinical-academic collaborations and appointments.^3^^,^^14^^,^^20^, ^21^, ^22^^,^^26^

### Limitations

While this study provides evidence to underpin the development of interventions to support capacity-building, resourcing, and enhance knowledge, attitudes and practice related to research in cancer nursing, the study is not without limitations. The generalizability of the results may be limited by the study setting and characteristics of the study sample. As a Magnet®-recognized cancer center, accreditation is in part dependent on ongoing scholarly work in the form of evidence-based practice implementation projects and new research.^38^ Our institution has invested in full- and part-time nurse scientist roles to support EBP and research efforts within the direct patient care setting, possibly influencing interest in and awareness of research practices. Furthermore, the majority of participants of this study were clinical nurses with 3 years or less of nursing experience, where interest in research may not yet be a priority.

## **Conclusions**

Within this study, nurses employed in a Magnet®-recognized cancer center report moderately positive research knowledge, willingness to engage in research and self-perceived ability to conduct research. However, the study has highlighted several factors that challenge the potential to build nursing research capacity, with barriers including time, incentivization, knowledge, and access to training and mentorship to support nursing research. Further education has the potential to improve knowledge and attitudes towards research; however, at an organizational level, investment will be required to ensure nurses have access to adequate infrastructure and resources and have scope and capacity within their roles to engage with and eventually lead clinical research programs. Initiatives that have the potential to ameliorate resource-based barriers are the formation of clinical research fellowships for nurses, buyout of clinical time, seed funding to develop research projects, and investment in human resources, which can support nurses’ development with regard to technical aspects of research processes, including protocol development, IRB approval processes, and data analysis skills.

## Ethics statement

The study was approved by the Institutional Review Board of the Memorial Sloan Kettering Cancer Center (IRB No. X18-003).

## Funding

This manuscript was funded in part through the NIH/NCI Cancer Center Support Grant P30 CA008748. The funders had no role in considering the study design or in the collection, analysis, interpretation of data, writing of the report, or decision to submit the article for publication.

## CRediT authorship contribution statement

BT was responsible for the concept and design of this study. BT and MBB collected the data. BT wrote the data analysis plan and analyzed the data, with support from AD and KF. All authors (AD, KF, PR, EL, MBB, BT) engaged in the interpretation of the data. AD prepared the draft manuscript. All authors (AD, KF, PR, EL, MBB, BT) critically reviewed and approved the paper. All authors had full access to all the data in the study, and the corresponding author had final responsibility for the decision to submit for publication. The corresponding author attests that all listed authors meet authorship criteria and that no others meeting the criteria have been omitted.

## Data availability statement

The datasets generated during and/or analyzed during the current study are not publicly available due to data protection and privacy concerns. Excerpts of data are available from the corresponding author upon reasonable request and following a signed data sharing agreement.

## Declaration of generative AI and AI-assisted technologies in the writing process

No AI tools/services were used during the preparation of this work.

## Declaration of competing interest

AD's work was supported in part by a Fulbright-Health Research Board Health Impact Award. AD is a Board Member of the European Oncology Nursing Society, and a Member of the European Commission Cancer Mission Board. KF, PR, EL, MBB and BT's work was supported in part by a Memorial Sloan Kettering Cancer Center Support Grant/Core Grant (Grant No. P30 CA008748) through funding from the National Cancer Institute. MBB serves on the editorial board of the Asia-Pacific Journal of Oncology Nursing. The article underwent standard review procedures of the journal, with peer review conducted independently of MBB and their research groups.
